# Canine Epithelial Thymic Tumors: Outcome in 28 Dogs Treated by Surgery

**DOI:** 10.3390/ani11123444

**Published:** 2021-12-02

**Authors:** Marina Martano, Paolo Buracco, Emanuela Maria Morello

**Affiliations:** 1 Department of Veterinary Medical Sciences, University of Parma, Strada del Taglio, 10, 43126 Parma, Italy; 2 Department of Veterinary Sciences, University of Torino, Largo P. Braccini 2, Grugliasco, 10095 Torino, Italy; paolo.buracco@unito.it (P.B.); emanuela.morello@unito.it (E.M.M.)

**Keywords:** dog, thymoma, canine thymic carcinoma, paraneoplastic *myasthenia gravis*, hypercalcemia of malignancy, thoracic surgery

## Abstract

**Simple Summary:**

Thymoma is a tumor of the cranial mediastinum rarely reported in dogs and should be differentiated from mediastinal lymphoma. CT and fine-needle aspirates or core biopsies are helpful in differential diagnosis, but flow cytometry may improve the pre-operative diagnostic ability. In thymomas, paraneoplastic syndromes such as *myasthenia gravis* and hypercalcemia may develop concurrently with the tumor. Their role as prognostic factors is not well determined. Surgical excision is the treatment of choice, but adjuvant radiotherapy and/or chemotherapy may prolong survival in cases of incomplete excision or if a thymic carcinoma is diagnosed. Local recurrence and metastasis are infrequently reported; therefore, a long survival is expected if the tumor is completely excised or if adjuvant therapy is undertaken. This article reports the authors’ experience with 28 dogs affected by 18 thymomas and 10 thymic carcinomas surgically treated from January 2000 to August 2021. The median overall survival time of the entire population was 1137 days; the median disease-free time was 903 days. Dogs with thymic carcinomas had significantly shorter disease-free intervals and shorter, although not statistically significant, survival times. Dogs with Masaoka Stage III tumors had worse outcomes.

**Abstract:**

Thymoma is a tumor rarely reported in dogs and should be differentiated from mediastinal lymphoma. Clinical signs may have a late onset, and thymoma is often diagnosed when symptoms related to the space-occupying effect or paraneoplastic syndromes occur. CT and fine-needle aspirates or core biopsies are helpful in differential diagnosis, but flow cytometry may improve the pre-operative diagnostic ability. Concurrent paraneoplastic syndromes such as *myasthenia gravis* and hypercalcemia have been reported; however, their role as prognostic factors is not well determined. Surgical excision is the treatment of choice; adjuvant radiotherapy and/or chemotherapy may prolong survival in cases of incomplete excision or when a thymic carcinoma is diagnosed. Local recurrence and metastasis are infrequently reported; therefore, a long survival time is expected if the tumor is completely excised or if adjuvant therapy is undertaken. This article reports the authors’ experience with 28 dogs affected by 18 thymomas and 10 thymic carcinomas. The median overall survival in this series was 1173 days, and the median disease-free interval was 903 days. Dogs with thymic carcinoma had significantly shorter disease-free intervals and shorter, although not statistically significant, survival times. Dogs with Masaoka Stage III tumors had worse outcomes.

## 1. Introduction

Thymoma is an uncommon canine tumor but is the second most common neoplasm of the cranial mediastinum of dogs, after lymphoma [1]. A similar incidence is reported in both cats and human beings, in which it represents up to 50% of cranial mediastinal tumors [2,3].

Clinical signs may be presented late, since they are mainly related to the space-occupying effect of the tumor itself; therefore, if they are not an incidental finding, diagnoses are often made when the mass is already of a relevant size, sometimes influencing the therapeutic options. In some instances, clinical signs related to paraneoplastic syndromes such as *myasthenia gravis* (MG) and hypercalcemia of malignancy are the complaints at presentation.

**Figure 1:**
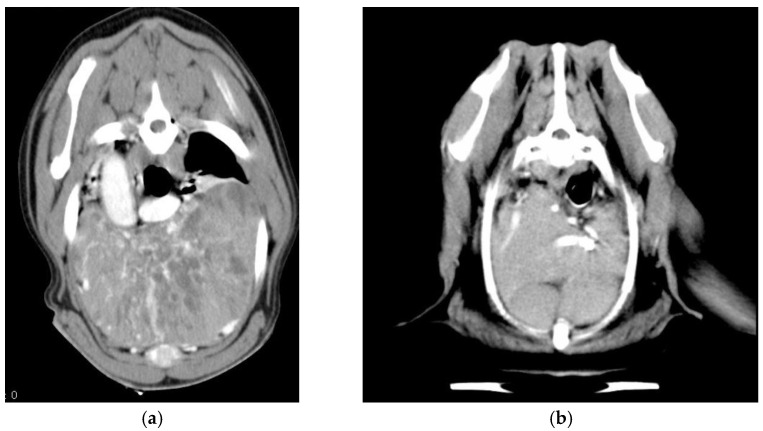
CT imaging of a large thymoma (**a**) and a lymphoma (**b**) occupying most of the cranial ventral mediastinum of two dogs. The heterogeneous, cystic appearance of the thymic tumor compared with lymphoma is evident.

One of the critical steps in the management of thymic epithelial tumors (TET)—as they are named in human medicine [4]—is definitive diagnosis, which requires histology in most cases, particularly for a differential diagnosis from mediastinal lymphoma. Imaging, cytology, and flow cytometry [5,6,7] may help in achieving the diagnosis, since some symptoms differ from those of mediastinal lymphoma, although this is not always straightforward (Figure 1).

Thymomas may have a more benign or malignant behavior depending on the type of growth—expansive or infiltrative—rather than on the histologic subtype; this characteristic influences invasion into surrounding tissues, such as the lungs, pericardium, and blood vessels, and thus the resectability of the mass.

The therapy of choice in both human and veterinary medicine is surgery [8,9,10,11,12]. If the thymoma can be completely removed and the capsule is not invaded, the prognosis is good [11].

Due to the low incidence of the tumor, only a few reports found in the literature—mainly multi-institutional—account for a large number of cases [7,8,9,10,13]. This case series represents the experience of the authors with canine TET in the last 20 years. A comparison with what has been reported in the literature within the same time interval is reported.

## 2. Materials and Methods

Dogs that underwent open thoracic surgery for the excision of a mass histologically diagnosed as a thymic epithelial tumor between January 2000 and August 2021 at the Veterinary Teaching Hospital of Grugliasco (Turin, Italy) were included in this study. The collected data included signalment, clinical (blood work and FNA biopsy of the mass), diagnostic imaging work up (thoracic radiographs, ultrasound, and/or CT of the chest), type of surgery, other therapies adopted, and the presence of paraneoplastic syndromes at diagnosis or developed during the follow-up. Dogs were included if a diagnosis of TET was histologically confirmed and they had a follow-up of at least 5 months. The histological diagnosis was based on the initial pathology report, which was made for the majority of cases by the same non-board-certified pathologist; no revision of the initial report was made by a board-certified pathologist.

During the time lapse considered, each dog admitted to the hospital for clinical signs related to the presence of a mediastinal or thoracic mass and/or of paraneoplastic syndromes related to mediastinal masses received a clinical examination, complete blood work, and three-view thoracic radiographs. Once presumptive diagnoses of the mediastinal mass had been achieved, an ultrasound-guided fine-needle aspiration of the mass was attempted. In more recent cases, a total body contrast-enhanced CT was also performed. When a definitive differential diagnosis from mediastinal lymphoma could not be reached by cytology and flow cytometry, exploratory thoracic surgery was performed.

Chemotherapy and radiotherapy (RT) were offered when a final diagnosis of thymic carcinoma was obtained, regardless of the completeness of excision. The chemotherapy drugs used included carboplatin, cyclophosphamide, and vincristine. The latter was mainly used in a neoadjuvant setting to achieve a cytoreduction in the tumor before surgery.

After surgery, the animals were hospitalized until the chest drain was in place, generally for 1 to 3 days.

The follow-up included a clinical examination, thoracic radiographs, and an evaluation of the signs of paraneoplastic syndromes every 4 months for the first year and every 6 months in the second year.

Data from long-term follow-up were obtained by phone calls to the owners or referring veterinarian in cases where they were not otherwise available.

The survival analysis was conducted using Kaplan–Meier’s product of survival probability curves. The log rank test was used to compare the survival curves. The analysis was conducted using the GraphPad 9 Prism program. The disease-free (DF) interval was considered the time from surgery to tumor recurrence or metastasis, and the overall survival (OS) time was calculated from the day of surgery to death from any cause. Dogs that died from non-tumor related causes, that were lost to follow-up, or that were alive at the end of the study were censored. Dogs that died within 1 week of surgery were considered perioperative deaths. Dogs that died because of paraneoplastic syndromes were considered as having died from the tumor. The canine population was divided into two groups based on a pathology report of thymoma or thymic carcinoma, and a survival analysis was conducted on the two populations. The same analysis was conducted on dogs divided according to Masaoka stages [14].

## 3. Results

### 3.1. Signalment and Clinical Findings

In this study, 28 dogs were included, of which 10 were males (one castrated) and 18 were females (seven spayed); the median age at presentation was 10 years (range: 6–13 years). Twelve dogs were mixed-breed animals, three were Rottweilers, two were Fox terriers, two were Labrador retrievers; and the number of Akita Inus, Alsatians (German shepherds), American Staffordshire terriers, Beagles, Dobermann pinschers, Golden retrievers, Lagottos, Shi-tzus, and Yorkshire terriers was one each. Some of these animals with thymic carcinoma were included in the study published by Yale et al. [9].

- *Diagnostics*: contrast-enhanced CT of the thorax without an angiogram was performed and available in 18 dogs; in older cases, medical records only reported three-view thoracic radiographs and thoracic ultrasounds for guidance in FNA of the mediastinal mass.
- *Paraneoplastic syndromes*: MG was diagnosed in two dogs (7.1%) before tumor removal and in three dogs (10.7%) after tumor excision, one of which was in the form of the focal megaesophagus; MG was confirmed by AchR antibody titers in four dogs and was presumed on the basis of the Tensilon test in one dog. The syndrome was resolved after surgery in one case and persisted in the other one. Pyridostigmine was administered to the animals in the generalized form. Hypercalcemia was diagnosed in two dogs (7.1%), both of which were affected by a thymic carcinoma, and one of which developed postoperative hypocalcemia that was resolved with symptomatic treatment after 15 days of hospitalization. The other dog presented with a large mediastinal mass and clinical signs of hypercalcemia, which was treated medically by the referring veterinarian with furosemide for 3 months before surgery was accepted by the owners. The syndrome was resolved after surgery, and it recurred at the time of tumor recurrence. Parathyroid hormone-related peptide (PTH-rp) was not evaluated in either animal.
  One dog was first seen for severe immune-mediated anemia that was treated by immunosuppressive doses of prednisone and cyclophosphamide and did not recur after extirpation of the tumor. The final diagnosis was thymoma, but the tumor recurred after 366 days and the dog was lost to follow up at that time.
- Five dogs (17.8%) had a concurrent tumor or developed one in the course of their life: one brochioloalveolar carcinoma, one lung squamous cell carcinoma, one squamous cell carcinoma of the digit, one cutaneous hemangiosarcoma, and one solid carcinoma of the frontal sinus. Brochioloalveolar carcinoma was the cause of death in one dog.

### 3.2. Therapy

**Figure 2:**
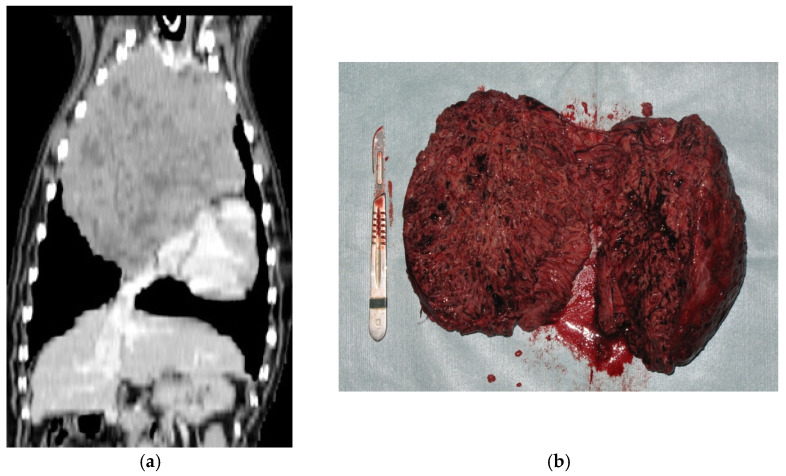
CT (**a**) and gross (**b**) appearance of a thymoma removed by sternotomy. The heterogeneity of the imaging is also visible macroscopically. The tumor could be removed en bloc.

Nineteen (65.5%) median sternotomies (Figure 2) and 10 (34.5%) intercostal thoracotomies were performed (one dog had a recurrence removed by median sternotomy after the excision of the primary tumor by the intercostal approach). Two dogs required a partial lung lobectomy to excise the tumor. 

Complications of the surgery included seroma formation in seven cases of median sternotomy, five of which required wound revision; fatal pulmonary thromboembolism in one case; and left subclavian artery rupture in one case, which was ligated and caused the dog to be lame on the left front leg for some days after surgery, which finally improved within 2 weeks. One dog died immediately after surgery because of heart failure, and one was euthanized intraoperatively because the tumor appeared to be non-resectable.

- *Neoadjuvant and adjuvant treatments*: chemotherapy was administered to seven dogs (25%), five postoperatively and two before surgery. The dog affected by immune-mediated anemia received cyclophosphamide (200 mg/m^2^/week) and prednisone (starting from 2 mg/kg/day for 1 week, tapering the dose in the following weeks) before surgery, which partially resolved the anemia. The other dog had an erroneous first diagnosis of lymphoma; therefore, a COP protocol (cyclophosphamide, vincristine, and prednisone) was started by the referring veterinarian, without success.
  The dog with hypercalcemia and thymic carcinoma received carboplatin (300 mg/m^2^) at the time of tumor recurrence, 60 days after surgery. A partial response was achieved, and the hypercalcemia was resolved. He is currently under treatment at the time of writing, but a complete response was not achieved, and the owners refused RT.

### 3.3. Histopathology Reports

Eighteen cases (64.3%) were diagnosed as thymoma (Types A, AB, and B1-2 of the WHO classification [15]), one (3.6%) as atypical thymoma (Type B3), and 10 (35.7%) as thymic carcinoma (Type C). Data on the completeness of excision were available in only five cases, of which three were completely excised; in two cases, the margins (the tumor capsule) were infiltrated, but only one recurred 60 days after surgery. In 25 dogs, the Masaoka staging system (Table 1) [14] could be retrospectively applied and resulted in eight Stage I, seven Stage II, and ten Stage III tumors. Metastases to regional lymph nodes were not detected in any of the excised nodes (five cases). Necropsy was performed only in one case of a dog that developed metastases 12 days after surgery and revealed metastasis to the liver, spleen, and pancreas.

### 3.4. Follow-Up

Seven dogs (25%) developed recurrence after 60 to 903 days (median: 60 days), two of which were diagnosed as thymoma. Two dogs (7.1%), one with a thymic carcinoma and one with a thymoma, developed metastasis after 289 and 12 days, respectively; the former developed lung metastasis diagnosed by thoracic radiographs, and the second developed metastasis to the abdominal organs.

One of the cases that developed a recurrence after 930 days from the first excision had a second surgery performed and survived for a total of 1340 days before dying spontaneously, probably due to the consequences of the megaesophagus and focal MG that developed 1 month before death.

According to the Kaplan–Meier survival curves, the median disease-free time of the entire population was 903 days (range: 0–2124 days) and the median overall survival time was 1137 days (range: 0–2124 days). Five dogs were alive at the end of the study after 145–809 days (median: 293 days). One of these dogs had a large thymic carcinoma with vascular invasion that had been incompletely resected and recurred macroscopically after 2 months; he is currently under carboplatin chemotherapy but not in complete remission. Three dogs (10.7%) died from causes not related to the tumor after 898, 912, and 2124 days; in particular, the causes of death were metastasis of a concurrent brochioloalveolar carcinoma, cardiomyopathy, and sudden death of unknown origin. Fourteen dogs (50%) died because of the tumor (four (14.3%) perioperatively) after 0 to 1340 days (median: 40.5 days), and six (21.4%) were lost to follow-up after 220–1358 days (median: 498.5 days), without signs of tumor recurrence or metastasis at the last follow-up.

When comparing dogs with thymic carcinoma (10 cases) against the ones with less aggressive histotypes, the DF interval was significantly shorter (*p* = 0.028) for thymic carcinomas (a median of 84.5 days vs. median not reached). Overall survival did not differ significantly (*p* = 0.076), although the median survival for thymic carcinomas was 291 days compared with the 1340 days achieved by less aggressive forms. The 1- and 2-year survival rates of the entire population were 58.3% and 38%, respectively.

**Figure 3:**
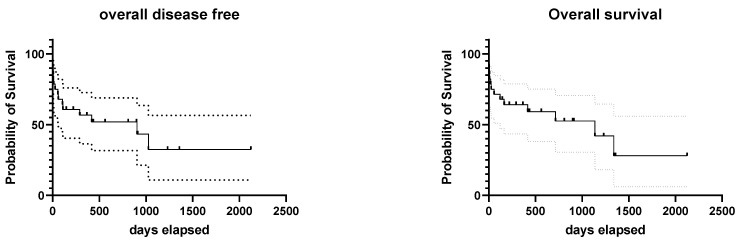
Kaplan–Meier survival curves showing the disease-free intervals and overall survival times of the 28 dogs, where the 95% confidence interval is shown by the dashed lines.

**Figure 4:**
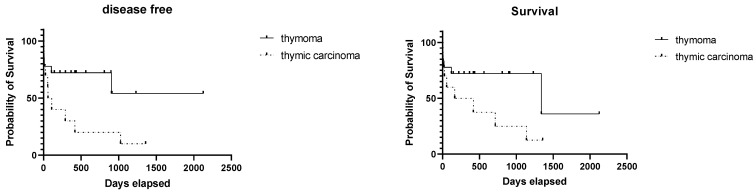
Kaplan–Meier survival curves showing the disease-free intervals and overall survival times of the dogs affected with thymoma and thymic carcinomas (dashed line). Although a difference between groups is evident in both graphs, the difference in OS is not statistically significant.

The Kaplan–Meier survival curves for the entire population and for the group of thymomas and thymic carcinomas are shown in Figure 3 and Figure 4.

**Figure 5:**
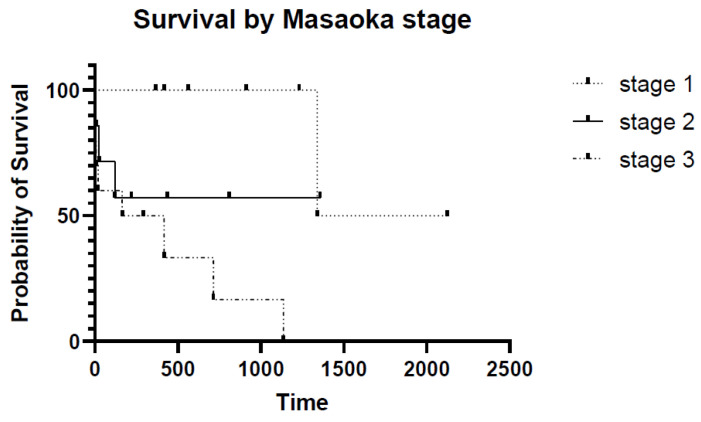
Kaplan–Meier survival curves of the 25 dogs divided into three subcategories based on Masaoka stage. A statistically significant difference was found in survival between dogs with Stage I and Stage III diseases.

When considering the Masaoka stage [14], the median survival of the eight Stage I dogs was 1732 days; two dogs were alive at the end of the study (after 145 and 417 days), and one dog died because of the tumor. Regarding the seven Stage II dogs, the median survival was not reached (mean: 424.8 days); one dog was alive after 809 days, and three died because of the tumor after 25, 120, and 220 days, respectively. Worse outcomes were observed in the 10 dogs with Stage III tumors, with a median survival of 291 days; eight dogs died from the tumor after 0 to 714 days, and two dogs are alive after 167 and 293 days. Table 2 shows the survival of dogs according to Masaoka stage, and Figure 5 shows the Kaplan–Meier survival curves of the three groups; the difference in survival is statistically significant between Stages I and III (*p* = 0.0049) but not between Stages II and III (*p* = 0.20).

MG was not resolved in two cases but was managed with lifelong pyridostigmine administration; hypercalcemia was resolved after surgery but recurred in the dog with thymic carcinoma at the time of tumor recurrence. The immune-mediated anemia did not recur after tumor extirpation, but the dog was lost to follow-up 366 days after surgery at the time of tumor recurrence.

## 4. Discussion

Thymoma is the second most common tumor of the cranial mediastinum in dogs, after lymphoma; nonetheless, it is infrequently reported [1]. Thymic tumors usually affect older animals, with a mean age of 9 to 10 years, although one case of a 1-year-old pug has been described [16]. There is no sex predilection, and some breeds seem to be overrepresented, such as Labrador retrievers, Alsatians, and Golden retrievers [2,7,8,10,13,17]. In the presented case series, the median age at presentation was 10 years, and no breed predilection was found except for mixed-breed dogs, which were the most represented.

Thymomas develop from the epithelial cells of the medulla of the thymus, but variable numbers of small lymphocytes may be present, thus making a differential diagnosis from lymphoma challenging by cytology alone. Small amounts of mast cells and eosinophils can also be observed. This difficulty may have been the cause of the initial diagnostic error that occurred in one case in this series. In fact, two main issues are present when differentiating thymomas from other mediastinal masses: the macroscopic differentiation by imaging from space-occupying masses, and microscopic differentiation from lymphoma before histology of the resected mass is performed. The differential diagnosis should be primarily versus mediastinal lymphoma, but other space-occupying masses should also be ruled out, such as branchial cysts, ectopic thyroid or parathyroid tumors, chemodectoma, thymic cysts, metastatic neoplasia, abscesses, granulomas, and other rarer thymus disorders such as thymolipoma and thymofibrolipoma [18,19,20,21].

Imaging is critical for evaluating the extent of the tumor, guide biopsies, and planning the surgery. The main preoperative concerns relate to the location of the mass (pulmonary vs. mediastinal), and its invasion into adjacent organs, especially the main blood vessels.

Three-view thoracic radiographs are the first step to determining the presence of a mediastinal mass and pleural effusion or displacement of the heart silhouette. In animals with megaesophagus, the enlargement of the organ, the ventral displacement of the trachea, or signs of aspiration pneumonia may be seen. The older cases in this series only had thoracic radiographs performed, and this may have been the cause of the difficulty in clearly defining the extent of the tumor before surgery.

Thoracic ultrasound is a further step that allows for better characterization of the mass and the performance of guided fine-needle or core biopsies [22]. FNA was always performed in this case series, although it did not always allow for differentiation from mediastinal lymphoma. Core biopsies have never been proposed due to the surgeon’s choice. In a study comparing 30 dogs and 5 cats with thymoma and 12 dogs and 3 cats with mediastinal lymphoma, Patterson and Marolf [23] found that echogenicity significantly differentiated the two tumor types, with thymoma being more heterogeneous compared with lymphoma.

Diagnostic imaging should be completed using computed tomography (CT), which is better for detecting the presence of metastases and for evaluating the size and extent of the tumor in the neighboring organs and vessels compared with radiographs [7,17,24]. CT was performed in 18 out of 28 dogs (64.3%) in this series, but an angiogram was never performed, which may have allowed us to identify the cases with larger blood vessel tumor involvement. In fact, three of the four perioperative deaths occurred in older cases that did not have CTs performed. Nevertheless, the perioperative mortality was not different from what has been reported by other authors [9,10]. Recent studies have reported better results for the ability of CT to detect vascular invasion [7] compared with older studies [24], although this parameter was not related to outcomes. Nevertheless, 5 of the 10 dogs with vascular invasion in the study of Scherrer and coworkers [24] died during the perioperative period. Both studies recommend venography during CT to better evaluate vessel involvement. Regarding vascular invasion, Reeve and colleagues [17] affirmed that the identification of vascular invasion was more difficult when a tumor thrombus was not present or when the vessel was markedly compressed by the mass itself. This fact should be considered when planning surgery on the basis of the CT results.

Pintore et al. [6] found a good correlation between cytological and histological evaluations of mediastinal masses in dogs and cats obtained by using either the blind technique, ultrasound, or CT, provided that the cytological samples were of good quality. Comparable results were found by Robat et al. [8], in which the cytology results were in agreement with the histology in 40 of 42 samples, and by Rick et al. [25], who found a concordance of 85%. To improve the ability of cytology in reaching a diagnosis, flow cytometry has been proposed as a valid approach to differentiating thymoma and lymphoma in dogs [5]. More than 80% of thymocytes express CD4 and CD8 markers, and this phenotype is different from other peripheral lymphocytes present in other organs. Therefore, it may be considered a way to differentiate thymoma from any other neoplastic masses in the cranial mediastinum in most cases. The presence of >10% of CD4+CD8+ cells in the small lymphocyte population was deemed 100% specific for thymoma in the study by Lana et al. [5]. As described for cytology, an appropriate sampling technique is important for retrieving a valuable sample. Nevertheless, two studies, by Burton and colleagues [26] and by Wikander and colleagues [27], reported a case of the CD4–CD8 double-negative immunophenotype of thymoma, which warns of the need to consider other diagnostic tools to prove or exclude the diagnosis of thymoma in such cases. In the cases presented here, flow cytometry was used only in two dogs, one of which led to the diagnosis of lymphoma for the first time and thymoma once the analysis was repeated.

Histology allows for a final diagnosis. In humans, thymic tumors are referred to as thymic epithelial tumors (TET), including both benign and malignant histotypes [4]. The most widely used staging system in humans is the Masaoka and Monden staging scheme [14], which considers tumor invasiveness as the main indicator of malignancy (Table 1). This scheme has been proposed to be also used by veterinary pathologists [8].

Canine TET staging may therefore refer to the Masaoka scheme [14], based on diagnostic imaging and the histological characteristics of the tumor, and to the modified WHO classification of the histologic grade [15,28]. In this case series, the Masaoka classification was applied retrospectively to cases where adequate information was available. This represents a limitation of the study, as is the lack of a revision of the histology by a board-certified pathologist. In fact, the occurrence of metastasis in one case diagnosed as thymoma may cause doubt regarding a misdiagnosis of this tumor. Moreover, the histology reports of older cases did not allow for an up-to-date classification into the different subtypes of thymoma, which were grouped as “thymoma”, unless a thymic carcinoma was diagnosed. The Masaoka classification revealed that Stage III tumors have a significantly worse prognosis compared with lower-stage tumors, in accordance to what has been found by Robat and coworkers [8].

The role of paraneoplastic syndromes in the clinical outcome is not fully understood, but aspiration pneumonia consequent to the presence of megaesophagus may worsen the prognosis. MG may develop either before or after surgery and does not always improve after tumor excision; therefore, the response of MG to medical treatments (pyridostigmine, corticosteroids, and other immunosuppressive drugs) may influence the long-term survival. However, its role as a negative prognostic factor is controversial, as reported by Zitz et al. [11], by Robat et al. [8], and by von Stade et al. [7]. In the present study, paraneoplastic MG was observed in a lower number of cases compared with what was reported by the literature (17.8% vs. 27–40%). In three cases, MG developed after tumor removal and needed to be managed for a variable period of time before resolution. In two cases, lifelong administration of pyridostigmine was necessary. The five dogs with MG survived for 56 to 1340 days (median: 220 days); therefore, no correlation with survival can be made. Nevertheless, complications of megaesophagus were the probable cause of death for the dog that developed it in the postoperative period.

Hypercalcemia of malignancy may be found in dogs with thymoma as well as in those with mediastinal lymphoma [29,30]. To confirm the paraneoplastic origin of this condition, in addition to a high ionized calcium, the blood level of PTH-rp should be high; in fact, the most accredited hypothesis considers the ectopic production of the parathyroid hormone as the cause of hypercalcemia in these cases [30]. Concurrently, a low parathyroid hormone blood level should be found. Two dogs in this series developed hypercalcemia of malignancy, which was not confirmed by PTH-rp evaluation. The paraneoplastic syndrome was resolved after tumor excision and recurred at the time of tumor recurrence.

Other hematological anomalies more rarely reported include anemia (immune-mediated or aplastic in humans [3]) (2016 Bernard Thymoma Associated with Autoimmune Diseases Autoimmunity Rev, n.d.), neutrophilia, lymphocytosis [26,31], thrombocytosis/thrombocytopenia, and erythema multiforme [32]. Myocarditis and polymyositis have also been reported [1]. Whether they are associated with a worse prognosis is still not well established [7,8,13,33], although more recent articles have reported that MG was associated with a shorter survival in dogs [9,10,34,35]. One dog in this series had anemia as a presenting clinical sign; the animal needed to be treated with immunosuppressive drugs before undergoing surgery, and the anemia improved significantly after tumor excision.

The treatment of choice in both human and veterinary medicine is surgery [8,9,10,11,12]. If the thymoma can be completely removed and the capsule is not invaded, the prognosis is good [11]. The limitations of resectability have changed over the years but mostly depend on the surgeon’s expertise in the field and confidence with this anatomic area, and on access to appropriate surgical instrumentation and anesthesia techniques. Perioperative mortality may be as high as 20% [10].The perioperative mortality in this study (14.3%) was a little lower than this but may have been due to the small number of dogs included and to selection bias relating to the retrospective nature of the study.

In a multi-institutional study conducted on 80 dogs and 32 cats, Garneau and coworkers [10] approached the tumor by median sternotomy in 90 cases; lung lobectomy was necessary in 21 dogs and four cats, whereas in four dogs, a section of the cranial vena cava wall was removed. In this study, potential risk factors for perioperative mortality were perioperative anemia and pleural effusion, while for long-term survival, perioperative anemia, paraneoplastic syndrome, and incomplete histologic margins were found to be prognostic. Tumor invasiveness was also considered to be a major prognostic factor for perioperative mortality in other studies [11] but not for long-term survival.

Depending on the size of the tumor, the surgical approach may be by lateral intercostal thoracotomy or median sternotomy. A major surgical complication of surgery is related to excessive bleeding from great vessel lacerations. Vessel laceration also occurred in one dog in this series, but it did not cause major complications. Seroma and suture dehiscence was the most common complication that occurred in dogs undergoing median sternotomy in this series, and required surgical revision in five of seven cases. This complication occurred in 36.8% of all sternotomies performed, which is a rather relevant incidence. No clinical factors could be found to be related to this complication, but the retrospective nature of the study limited this analysis.

Tumor recurrence was reported in 17% [8] of cases; a second surgery could be performed in most cases, leading to a prolonged survival time. In this series, the recurrence rate was 25%; in one case, a second surgery was performed, and the dog had a long survival (1340 days), which is in line with what has been reported in the literature. The higher recurrence rate may be due to the size of the tumors, which was large in many cases, thus limiting the possibility of complete excision.

Metastases may occur in regional intrathoracic lymph nodes and the lungs in up to 20% of cases [1] but are rarely diagnosed at presentation. Rare reports of metastasis to other organs, such as the liver, have been reported [36]. One dog in this study developed liver metastasis, which was diagnosed at necropsy; this dog had a diagnosis of thymoma, but this finding may raise the suspicion of a diagnostic error.

In the case of large or incompletely excised tumors, radiation therapy and/or chemotherapy may be performed in an adjuvant setting. Thymoma is considered a radiosensitive tumor, especially the lymphocyte-rich subtype [33], and different protocols have been proposed [33,37,38,39]. RT has been proposed as a monotherapy with a palliative intent in inoperable tumors. In this case, the control of acute and late side effects is essential for quality of life reasons. A hypofractionated protocol has been investigated with this aim [38]. RT was proposed in the case of thymic carcinoma in the present study, regardless of the completeness of excision, but was declined by all owners. This therapy could have made some difference in the outcome in those cases, but the paucity of the facilities and the high costs still limit its use, at least in some countries.

The role of chemotherapy in the treatment of canine thymoma is not well established yet, since no prospective clinical trials have investigated this subject. In human medicine, chemotherapy is proposed in cases that are inoperable, at an advanced stage, or have recurrent tumors [40]. The protocols more commonly used are based on platinum–anthracycline, with or without etoposide, or platinum and taxanes [12,40]. Targeted therapies do not seem to be effective, while immunotherapy with anti PD1/PDL-1 has produced promising results [40]. In dogs, chemotherapy has always been used as an adjuvant to surgery or RT [8,9,10,11]; the most commonly used agents are carboplatin, doxorubicin, cyclophosphamide, vincristine, toceranib phosphate, and prednisone, alone or in different combinations, or metronomic therapy. The results of the treatment are variable, and too few cases have been reported to draw any reliable conclusions, although the same indication as for human beings should probably be followed. Chemotherapy was administered in a few cases, with different drugs (carboplatin, cyclophosphamide, and vincristine) and protocols in this study; therefore, nothing can be said about its efficacy. The adjuvant administration of carboplatin in a prospective study may be worth evaluating.

In some cases, a second non-thymic tumor may be diagnosed concurrently [8]. This concurrence has also been described in humans, where a second non-thymic tumor may develop in 8 to 28% of cases [4]. A second tumor developed in five dogs (17.8%) in this study, concurrently or not with the thymoma. This event does not seem to have influenced survival in this case series.

The prognosis for thymoma in dogs is mainly associated with the completeness of excision, the invasion of the capsule, and the survival to surgery (because of perioperative complications) [7,8,9,10,11]. Masaoka Stages III and IV but not histologic subtypes were considered a negative prognostic factor in Robat and coworkers’ study [8]. Additionally, in this case series, Masaoka Stage III had a significantly shorter survival (median: 291 days) compared with Stage I tumors (median: 1732 days).

Table 3 shows a summary of the outcomes of the cases included.

## 5. Conclusions

The small case series reported here is in line with what was previously described in terms of the incidence of paraneoplastic syndromes and outcomes. Thymic carcinoma was diagnosed in 10 cases (35.7%), a rather relevant percentage; nevertheless, two dogs survived for 1137 and 714 days, and one was still alive after 167 days from surgery and 254 days from the initial diagnosis, meaning that, if surgical excision/debulking is feasible, long-term survival may also be achievable for these dogs. Adjuvant RT may have improved these data, but it was never accepted by owners. The lower mortality rate reported in this series compared with that in the literature may be due to selection bias and the low case numbers. 

In conclusion, thymoma is a rare tumor in dogs that should be differentiated from other space-occupying masses of the cranial mediastinum. Bulky tumors may be surgically excised or debulked, and this can prolong survival, especially when adjuvant radiotherapy is associated, while the role of chemotherapy is not completely defined.

Additionally, the resection of a recurring tumor may prolong survival. Paraneoplastic syndromes usually regress after treatment; nonetheless, MG may not be resolved or even develop after tumor removal but it could be managed medically. Megaesophagus may be a more severe consequence to consider.

DF and OS were longer than those reported by the literature, but this could be due to the small number of cases and/or selection bias. Additionally, it may support the conclusion that when surgical excision is possible and regular restaging of the thorax is performed, long-term survival may be achieved.

## Figures and Tables

**Table 1:** The Masaoka staging system [14].

Stage	Description
I	Completely encapsulated
II	Invades the capsule, either microscopically or macroscopically
III	Invades the neighboring organs, with or without great vessel involvement
IV	Metastatic, either locally on the thoracic mesothelium or distantly

**Table 2:** Survival of dogs according to the Masaoka stage [14].

Stage	N. of Cases	Median Survival (Days)	Alive	Dead from the Tumor	Lost to Follow-Up/Dead from Unrelated Causes
I	8	1732	2	1	4
II	7	n.r.	1	3	3
III	10	291	2	8	0
IV	0	-	-	-	-

n.r. = not reached.

**Table 3:** Outcome data of the 28 dogs included in the authors’ case series.

Case #	Breed	CT	Paraneoplastic HC ^1^/MG ^2^	Masaoka Stage	Histotype	Recurrence (R) Metastasis (M)	DF ^3^ (Days)	OS ^4^ (Days)	Status
**1**	Mix	no		n.d. ^5^	Thymoma		898	898	Dead * ^6^
**2**	Mix	no		III	Invasive thymoma		0	0	Dead
**3**	Mix	no		I	Thymoma	R	366	366	LFU
**4**	Rottweiler	yes		n.d.	Thymoma		8	8	Dead
**5**	Mix	no		II	Thymoma	R	105	120	Dead
**6**	Rottweiler	no		I	Thymoma		912	912	Dead *
**7**	Labrador retriever	no	MG	I	Thymoma	R	903	1340	Dead
**8**	Alsatian (German shepherd)	no		III	Thymoma	M	12	20	Dead
**9**	Fox terrier	no		I	Thymoma		562	562	LFU
**10**	Mix	Yes	HC	II	Thymic k ^7^		1358	1358	LFU
**11**	Mix	Yes		III	Thymic k	R	417	417	Dead
**12**	Shi-tzu	No		III	Thymoma		0	0	Dead
**13**	Mix	Yes		II	Thymic k		7	7	Dead
**14**	Mix	Yes		I	Thymoma		1231	1231	LFU
**15**	Mix	Yes	MG	n.d.	Thymic k		56	56	Dead
**16**	Akita Inu	Yes		I	Thymoma		2124	2124	Dead *
**17**	Beagle	Yes	MG	III	Thymic k	R	1025	1137	Dead
**18**	Labrador retriever	Yes		III	Thymic k	R/M	289	714	Dead
**19**	Golden retriever	Yes		II	Thymoma		435	435	LFU
**20**	Mix	Yes		II	Thymic k		25	25	Dead
**21**	Fox terrier	Yes		II	Thymoma		809	809	Alive
**22**	Lagotto	Yes		I	Thymoma		417	417	Alive
**23**	American Staffordshire terrier	No	MG	II	Atypical thymoma		220	220	LFU
**24**	Yorkshire terrier	Yes		III	Thymoma		293	293	Alive
**25**	Doberman pinscher	Yes	MG	I	Thymoma		145	145	Alive
**26**	Rottweiler	Yes	HC	III	Thymic k	R	60	167	Alive
**27**	Mix	Yes		III	Thymic k		7	7	Dead
**28**	Mix	Yes		III	Thymic k	R	109	165	Dead

^1^ hypercalcemia; ^2^ *myastenia gravis*; ^3^ disease-free; ^4^ survival; ^5^ not determined; ^6^ dead from causes not related to the tumor; ^7^ carcinoma.

## Data Availability

The data are contained within the article.
